# Differential serum microRNAs in premotor *LRRK2* G2019S carriers from Parkinson’s disease

**DOI:** 10.1038/s41531-023-00451-x

**Published:** 2023-02-02

**Authors:** Marta Soto, Manel Fernández, Paloma Bravo, Sara Lahoz, Alicia Garrido, Antonio Sánchez-Rodríguez, María Rivera-Sánchez, María Sierra, Paula Melón, Ana Roig-García, Anna Naito, Bradford Casey, Jordi Camps, Eduardo Tolosa, María-José Martí, Jon Infante, Mario Ezquerra, Rubén Fernández-Santiago

**Affiliations:** 1 Lab of Parkinson Disease and Other Neurodegenerative Movement Disorders, Institut d’Investigacions Biomèdiques August Pi i Sunyer (IDIBAPS), Institut de Neurociències, Universitat de Barcelona, ES-08036 Barcelona, Catalonia Spain; 2 Parkinson Disease and Movement Disorders Unit, Neurology Service, Institut Clínic de Neurociències, Hospital Clínic de Barcelona, ES-08036 Barcelona, Catalonia Spain; 3 Centro de Investigación Biomédica en Red sobre Enfermedades Neurodegenerativas (CIBERNED: CB06/05/0018-ISCIII), ES-08036 Barcelona, Catalonia Spain; 4 Parkinson’s Disease and Movement Disorders Group of the Institut de Neurociències (Universitat de Barcelona), ES-08036 Barcelona, Catalonia Spain; 5 Gastrointestinal and Pancreatic Oncology Team, Institut d’Investigacions Biomèdiques August Pi i Sunyer (IDIBAPS)-Hospital Clínic de Barcelona, 08036 Barcelona, Spain; 6 Centro de Investigación Biomédica en Red de Enfermedades Hepáticas y Digestivas (CIBERehd), Madrid, Spain; 7 Movement Disorders Unit, Department of Neurology, Hospital Universitario Marqués de Valdecilla, Universidad de Cantabria, ES-39008 Santander, Cantabria Spain; 8 The Michael J. Fox Foundation for Parkinson’s Research, Grand Central Station, P.O. Box 4777, New York, NY 10120 USA; 9 Histology Unit, Department of Biomedicine, Faculty of Medicine, Universitat de Barcelona, ES-08036 Barcelona, Catalonia Spain

**Keywords:** Diagnostic markers, Parkinson's disease, Movement disorders, Neurodegenerative diseases, Biomarkers

## Abstract

The *LRRK2* G2019S pathogenic mutation causes LRRK2-associated Parkinson’s disease (L2PD) with incomplete penetrance. LRRK2 non-manifesting carriers (L2NMC) are at PD high risk but predicting pheno-conversion is challenging given the lack of progression biomarkers. To investigate novel biomarkers for PD premotor stages, we performed a longitudinal microRNA (miRNA) assessment of serum samples from G2019S L2NMC followed-up over 8 years. Our cohort consisted of G2019S L2NMC stratified by dopamine transporter single-photon emission computed tomography (DaT-SPECT) into DaT-negative (*n* = 20) and DaT-positive L2NMC (*n* = 20), pheno-converted G2019S L2PD patients (*n* = 20), idiopathic PD (iPD) (*n* = 19), and controls (*n* = 40). We also screened a second cohort of L2PD patients (*n* = 19) and controls (*n* = 20) (Total *n* = 158). Compared to healthy controls, we identified eight deregulated miRNAs in DaT-negative L2NMC, six in DaT-positive L2NMC, and one in L2PD. Between groups, the highest miRNA differences, 24 candidate miRNAs, occurred between DaT-positive L2NMC and L2PD. Longitudinally, we found 11 common miRNAs with sustained variation in DaT-negative and DaT-positive L2NMCs compared to their baselines. Our study identifies novel miRNA alterations in premotor stages of PD co-occurring with progressive DaT-SPECT decline before motor manifestation, whose deregulation seems to attenuate after the diagnosis of L2PD. Moreover, we identified four miRNAs with relatively high discriminative ability (AUC = 0.82) between non-pheno-converted DaT-positive G2019S carriers and pheno-converted L2PD patients (miR-4505, miR-8069, miR-6125, and miR-451a), which hold potential as early progression biomarkers for PD.

## Introduction

Parkinson’s disease (PD) is an age-related neurodegenerative movement disorder^1^ which is characterized by the loss of dopaminergic neurons (DAn) in the substantia nigra pars compacta and Lewy bodies containing α-synuclein in different brain areas^2,3^. The clinical diagnosis is based on motor symptoms (bradykinesia, rigidity, and resting tremor). Still, prodromal disease stages may course with non-motor symptoms such as REM sleep behavior disorder (RBD), hyposmia, constipation, or depression^4,5^. Most PD cases are classified as idiopathic PD (iPD) patients, but 5–10% encompass monogenic forms^6^. Among these, mutations in the leucine-rich repeat kinase 2 gene (*LRRK2*) leading to LRRK2-associated PD (L2PD) are the most frequent monogenic cause of PD^7,8^. However, the penetrance of mutations in *LRRK2*, e.g., G2019S, is incomplete and varies across populations, suggesting the involvement of additional modulators^9–13^. Thus, LRRK2 non-manifesting carriers (L2NMC) are at a higher risk of PD, but predicting disease onset is challenging due to the lack of early progression biomarkers.

MicroRNAs (miRNAs) are small non-coding RNAs regulating gene expression by mRNA cleavage and translational repression^14^. MiRNA alterations have been associated with PD^15,16^, and some miRNAs have been proposed as non-invasive candidate biomarkers^17,18^. However, longitudinal studies are needed, especially in PD at-risk subjects. Thus, to explore early progression biomarkers, we profiled serum miRNA expression levels in a cohort of G2019S L2NMC from Spain, which we followed-up over 8 years^19,20^. Given that dopamine transporter single-photon emission computed tomography (DaT-SPECT) correlates with striatal PD DAn loss^21,22^ and that L2NMC can show reduced striatal ligand uptake before the onset of the motor symptoms^23^, we stratified our cohort into DaT-positive and DaT-negative G2019S L2NMC^20,24^. By genome-wide miRNA discovery (2578 miRNAs) and RT-qPCR validation, we investigated differentially expressed miRNAs (DEmiR) in G2019S L2NMC, L2PD patients, iPD, and healthy controls^20^. Overall, this study tackles early miRNA deregulation at PD premotor stages and evaluates the potential of miRNAs as candidate progression biomarkers for PD.

## Results

### Cross-sectional genome-wide miRNA analysis

**Fig. 1:**
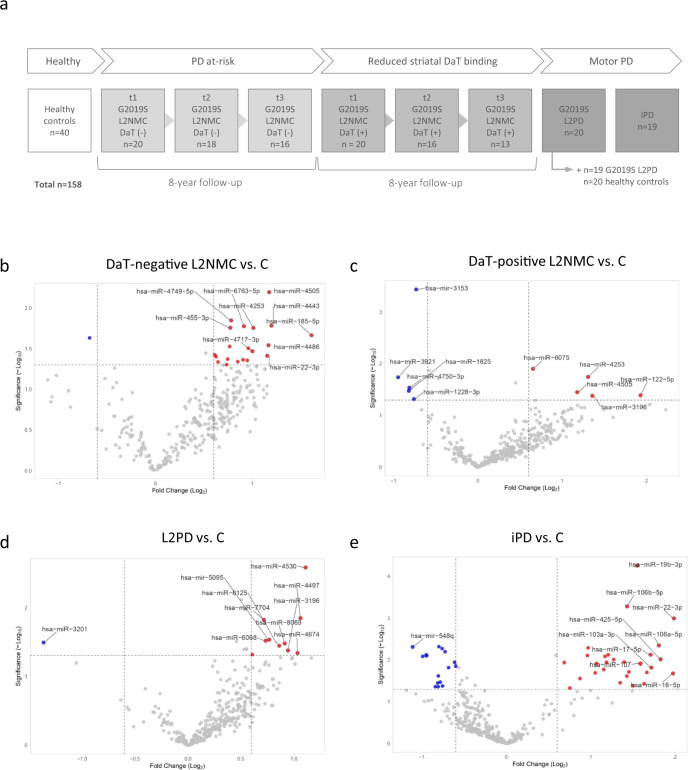
Workflow of the study and differential miRNA expression in DaT-negative L2NMC, DaT-positive L2NMC, L2PD patients, and iPD patients by genome-wide miRNA analysis in the discovery phase. **a** Workflow of the study and number of participating subjects. **b** Volcano plot of differential miRNA expression between DaT-negative L2NMC and controls, **c** DaT-positive L2NMC and controls, **d** L2PD and controls, and **e** iPD and controls. Candidate differentially expressed miRNAs were defined as miRNAs with a fold-change above |1.5| and a *p* value below 0.05 under a two-tailed Student’s *t* test. Only names of the top-10 miRNAs are shown. Up-regulated and down-regulated DEmiR are respectively depicted in red and blue. DaT DaT-SPECT imaging, L2NMC LRRK2 non-manifesting carriers, L2PD LRRK2-associated PD patients, iPD idiopathic PD patients.

**Fig. 2:**
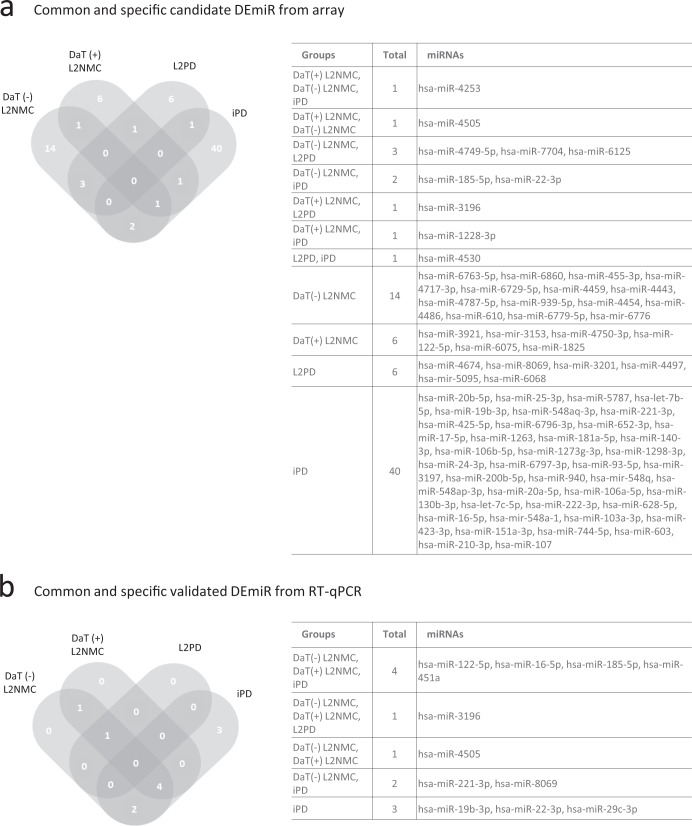
Common and specific candidate DEmiR of DaT-negative L2NMC, DaT-positive L2NMC, L2PD, and iPD as compared to controls. Venn diagram of **a** candidate DEmiR from the discovery analysis and **b** differentially expressed miRNAs (DEmiR) from the RT-qPCR miRNA assessment. DaT DaT-SPECT imaging, L2NMC LRRK2 non-manifesting carriers, L2PD LRRK2-associated PD patients, iPD idiopathic PD patients.

In our cohort of study (Table 1 and Fig. 1a), by genome-wide discovery analysis and under a *p* value below 0.05 and a fold-change above |1.5|, we identified 21 candidate miRNAs in DaT-negative L2NMC vs. controls and 10 in DaT-positive L2NMC (Fig. 1b, c). We also found 11 candidate miRNAs in G2019S L2PD and 45 in iPD compared to controls. These findings indicate a more pronounced miRNA deregulation effect in iPD than L2PD (Fig. 1d, e and Supplementary Table 1A). In addition, across all four comparisons, we observed an overlap in two or more groups of only 10 (13%) out of the 76 unique miRNAs, thus illustrating that most of the deregulated miRNAs identified at the genome-wide level were group-specific (Fig. 2a). Moreover, in line with previous findings, in iPD cases we found miR-19b-3p as the top deregulated miRNA by the array^25,26^. Altogether, these results indicate that microRNA deregulation is more prominent in iPD than in L2PD and that specific miRNA changes occur across the continuum of progression stages in G2019S carriers including DaT-negative and DaT-positive L2NMC.

**Table 1:** Clinico-demographic data of participants.

Total *n* = 158	Nr. of studied subjects	Sex (M/F)	Age at PD onset ± SD (years)	Age at sampling ± SD (years)	Time from baseline ± SD (years)	Mean LEDD ± SD (mg)	Mean MDS UPDRS-III ± SD (Nr. of available from total)	Nr. of pheno-converted subjects
Controls	40	28/12	–	65.48 ± 11.69	–	–	–	–
L2NMC DaT-	20	8/12	–	52.30 ± 10.12	–	–	0.39 ± 0.61 (*n* = 18/20)	–
Time point 2	18	6/12	–	56.78 ± 11.60	4.32 ± 1.68	–	1.72 ± 1.79 (*n* = 16/18)	0
Time point 3	16	7/9	–	59.50 ± 11.40	7.79 ± 2.22	–	2.33 ± 2.23 (*n* = 12/16)	0
L2NMC DaT+	20	12/8	–	60.50 ± 14.49	–	–	3.10 ± 5.22 (*n* = 20/20)	–
Time point 2	16	9/7	–	61.63 ± 13.26	5.02 ± 1.50	–	6.34 ± 10.26 (*n* = 16/16)	3
Time point 3	13	8/5	–	63.85 ± 11.63	9.12 ± 1.68	–	5.19 ± 5.64 (*n* = 8/13)	4
L2PD	20	12/8	58 ± 13.14	65 ± 10.90	–	802.47 ± 810.02	22.00 ± 13.10 (*n* = 17/20)	–
iPD	19	12/7	56.16 ± 13.01	63.53 ± 11.77	–	420.13 ± 391.46	25.00 ± 16.37 (*n* = 8/19)	–
L2PD 2nd cohort	19	8/11	53.47 ± 10.79	64.47 ± 11.34	–	708.68 ± 469.64	28.05 ± 13.87 (*n* = 19/19)	–
Controls 2nd cohort	20	8/12	–	63.65 ± 10.75	–	–	–	–

UPDRS-III scaling is provided as a mean ± standard deviation (SD) with the number of subjects with available data specified in brackets.

*DaT* DaT-SPECT imaging, *L2NMC* LRRK2 non-manifesting carriers, *L2PD* LRRK2-associated PD patients, *iPD* idiopathic PD patients, sex: *M* males, *F* females, *LEDD* levodopa equivalent daily dose, *MDS UPDRS-III* Unified Parkinson’s Disease Rating Scale scoring of the Movement Disorders Society.

### Cross-sectional RT-qPCR

**Fig. 3:**
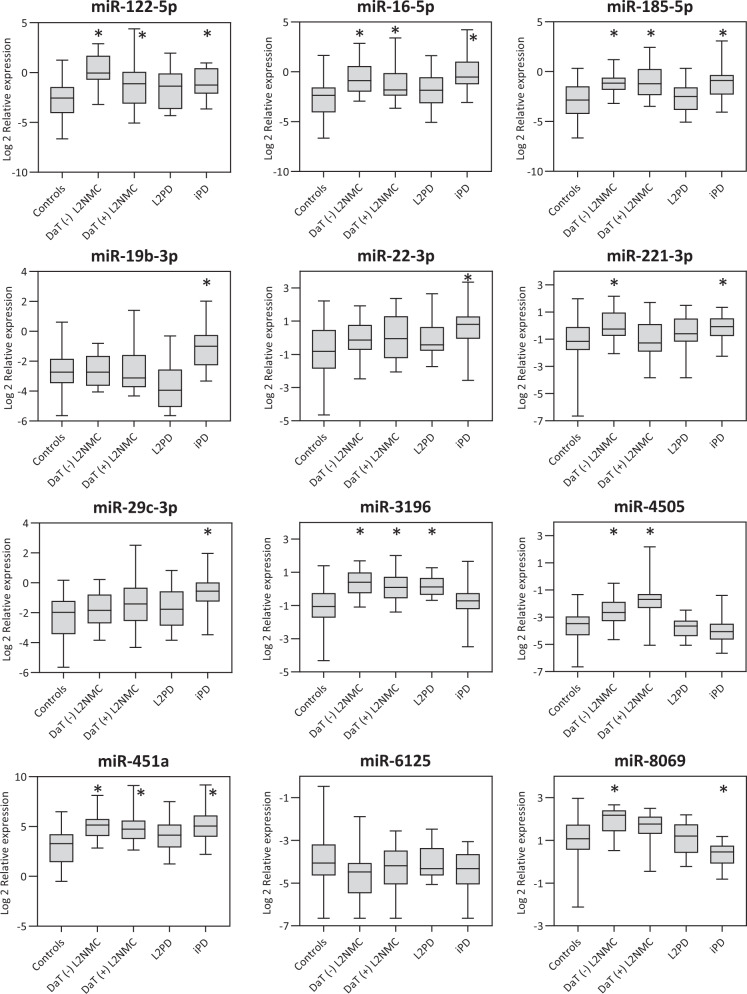
Relative serum miRNA expression levels in DaT-negative L2NMC, DaT-positive L2NMC, L2PD, iPD and controls as assessed by RT-qPCR. The central line of the boxes is plotted at the median, the boxes extend from the 25th to the 75th percentiles, and the whiskers extend to the minimum and maximum value. (*) depicts statistically significant DEmiR with fold-change difference above |1.5| and a multiple-test adjusted *p* value below 0.05 under two-tailed Student’s *t* test. DaT DaT-SPECT imaging, L2NMC LRRK2 non-manifesting carriers, L2PD LRRK2-associated PD patients, iPD idiopathic PD patients.

By RT-qPCR, we assessed the expression levels of ten candidate miRNAs found in the genome-wide analysis across different comparisons (Supplementary Table 2) and two additional candidate miRNA earlier reported in iPD (miR-29c-3p)^25,27^ or PD prodromal stages (miR-451a)^28^. Using the same serum samples, we validated 8 DEmiR, which were deregulated in one or more G2019S carrier groups (miR-122-5p, miR-16-5p, miR-185-5p, miR-221-3p, miR-3196, miR-4505, miR-451a, and miR-8069) (Fig. 3 and Table 2). Of these, all eight miRNAs were up-regulated in DaT-negative L2NMC, six in DaT-positive L2NMC (miR-122-5p, miR-16-5p, miR-185-5p, miR-3196, miR-4505 and miR-451a), and only one in L2PD (miR-3196). Thus, miR-3196 was the only DEmiR common for all G2019S carriers (Fig. 2b). Moreover, to confirm findings in L2PD, we tested an independent set of L2PD patients and controls of equal size (Table 1) and, in line with the initial results, we observed few differences between L2PD and controls (miR-122-5p) (Supplementary Table 3). Although miR-122-5p but not miR-3196 was the miRNA detected in the validation set, given our limited size, both miRNAs represent proposed candidates for subsequent studies in larger L2PD cohorts. Altogether, these results support the concept of a progressive decline in the number of miRNAs deregulated across the successive progression stages in G2019S carriers. In addition, in iPD cases, we found 9 of the 12 studied miRNAs deregulated (miR-122-5p, miR-16-5p, miR-185-5p, miR-19b-3p, miR-22-3p, miR-221-3p, miR-29c-3p, miR-451a, and miR-8069), again detecting greater miRNA deregulation in iPD than in L2PD. Lastly, miR-19b-3p and miR-29c-3p, which were earlier reported as DEmiR in iPD and idiopathic RBD^25–27,29,30^, showed significant changes only in iPD but not in L2PD nor L2NMC, thus indicating differential miRNA deregulation between iPD and the continuum of progression stages in G2019S carriers, at least for these two specific miRNAs.

**Table 2:** RT-qPCR assessment of miRNA levels in serum samples from DaT-negative and DaT-positive L2NMC, L2PD patients and iPD as compared to healthy controls.

MicroRNA	DaT (−) L2NMC (*n* = 20)		DaT (+) L2NMC (*n* = 20)		L2PD (*n* = 20)		iPD (*n* = 19)	
	FC	Adj. *p*	FC	Adj. *p*	FC	Adj. *p*	FC	Adj. *p*
miR-122-5p	7.07	3.0 × 10^−6^	3.38	0.0269	2.01	0.1616	3.05	0.0025
miR-16-5p	3.42	0.0007	2.51	0.0237	1.66	0.2590	5.21	0.0003
miR-185-5p	3.68	3.0 × 10^−5^	4.32	0.0004	1.49	0.3122	3.60	0.0028
miR-19b-3p	1.05	0.8394	1.25	0.5280	−2.05	0.1303	3.04	0.0018
miR-22-3p	1.66	0.0632	1.77	0.0759	1.64	0.1611	2.67	0.0025
miR-221-3p	2.06	0.0079	1.15	0.6263	1.37	0.3122	1.93	0.0076
miR-29c-3p	1.44	0.1435	1.93	0.0759	1.45	0.2741	2.67	0.0025
miR-3196	2.43	1.0 × 10^−5^	1.98	0.0012	2.13	0.0001	1.11	0.6142
miR-4505	1.98	0.0016	3.51	0.0004	−1.06	0.7803	−1.28	0.3225
miR-451a	4.44	1.1 × 10^−5^	4.16	0.0004	2.43	0.0629	4.76	0.0016
miR-6125	−1.47	0.1179	−1.26	0.3307	−1.01	0.9535	−1.32	0.2461
miR-8069	1.89	0.0006	1.44	0.0759	1.09	0.7352	−1.53	0.0145

*DaT* DaT-SPECT imaging, *L2NMC* LRRK2 no manifesting carrier, *L2PD* LRRK2 carrier with symptomatic Parkinson disease, *iPD* idiopathic Parkinson disease, *FC* fold change, *Adj. p* FDR multiple-test adjusted *p* value.

### Comparison of progression stages in G2019S carriers

**Fig. 4:**
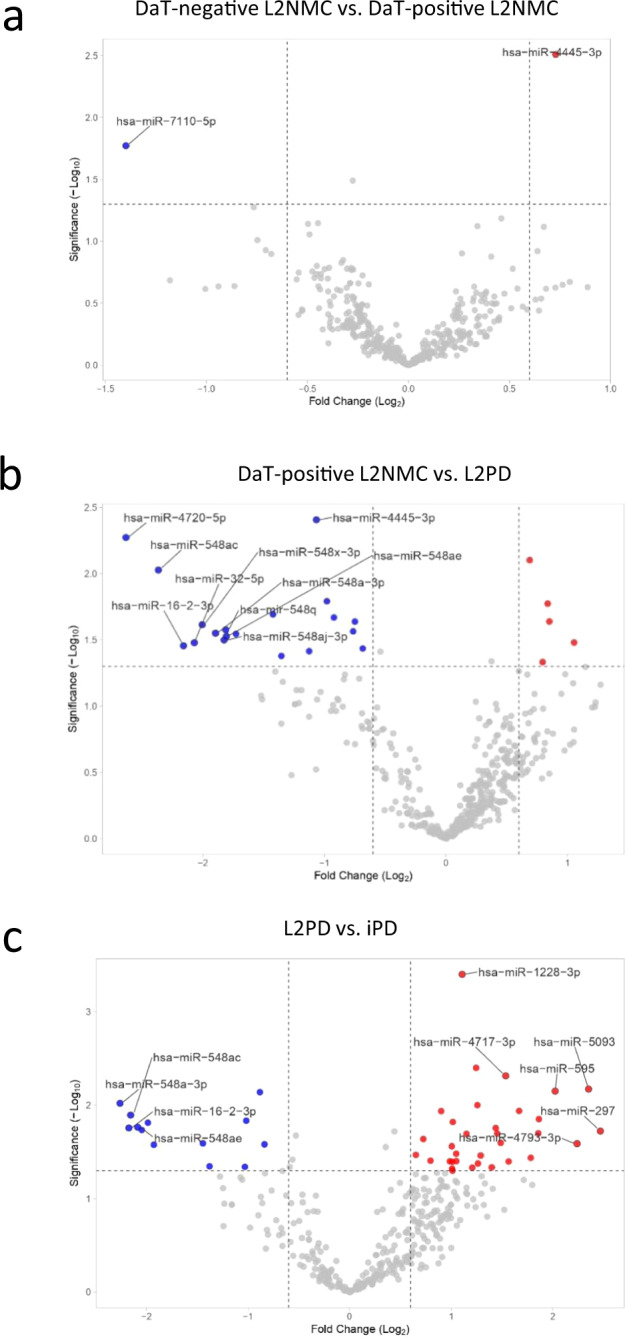
Differential miRNA expression in successive G2019S LRRK2 progression stages and iPD. Volcano plots showing miRNA expression differences between **a** DaT-negative and DaT-positive L2NMC, **b** DaT-positive L2NMC and L2PD, and **c** L2PD and iPD patients. DEmiR were defined as miRNAs with a fold-change above |1.5| and a *p* value below 0.05 under a two-tailed Student’s *t* test. Only names of the top-10 miRNAs are shown. Up-regulated and down-regulated DEmiRs are respectively depicted in red and blue. DaT DaT-SPECT imaging, L2NMC LRRK2 non-manifesting carriers, L2PD LRRK2-associated PD patients, iPD idiopathic PD patients.

**Fig. 5:**
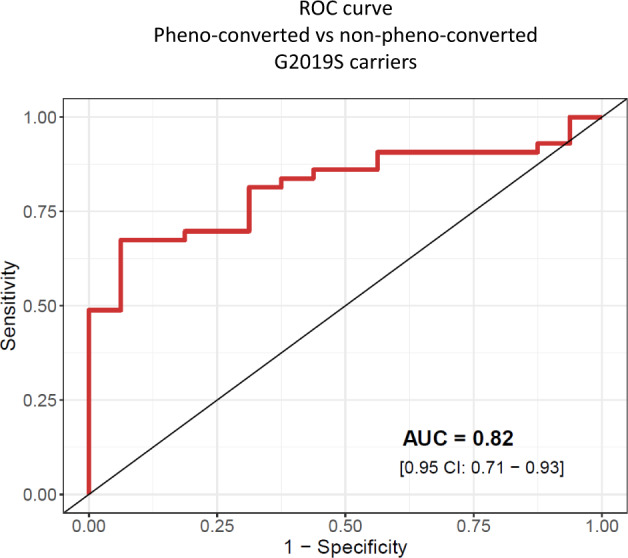
Area under the curve (AUC) analysis on a receiver operating characteristic (ROC) curve representing the discriminative ability of four miRNAs associated with pheno-conversion (miR-4505, miR-8069, miR-6125, and miR-451a) when comparing DaT-positive L2NMC non-pheno-converted after 8 years of follow-up (*n* = 16) vs. all pheno-converted subjects, i.e., L2PD discovery (*n* = 20) and validation (*n* = 19) sets, and DaT-positive L2NMC diagnosed with PD during the study (*n* = 4) (total *n* = 43). DaT DaT-SPECT imaging, L2NMC LRRK2 non-manifesting carriers, L2PD LRRK2-associated PD patients.

We next inquired about miRNA differences occurring across the continuum of progression stages in G2019S carriers. To this end, using genome-wide miRNA data, we first performed pair-wise comparisons among DaT-negative L2NMC, DaT-positive L2NMC, and L2PD (Fig. 4a–c and Supplementary Table 1B). We found modest miRNA changes between DaT-negative and DaT-positive L2NMC despite their different DaT-SPECT status (miR-7110-5p and miR-4445-3p). However, we observed that most miRNA differences occurred between DaT-positive L2NMC and L2PD patients with overt disease (24 miRNAs), thus suggesting specific miRNA deregulation occurring along with changes in disease status. Moreover, we exploratorily assessed potential miRNA changes related to pheno-conversion by comparing DaT-positive L2NMC non-pheno-converted after 8 years of follow-up (*n* = 16) vs. all pheno-converted subjects, i.e., L2PD discovery (*n* = 20) and validation (*n* = 19) sets, and DaT-positive L2NMC which pheno-converted during the study (*n* = 4) (total *n* = 43). We identified four miRNAs significantly associated with pheno-conversion including miR-4505 (adj. *p* = 0.0006), miR-8069 (adj. *p* = 0.0035), and miR-6125 (adj. *p* = 0.0280) and a borderline trend for miR-451a (adj. *p* = 0.0828) (Supplementary Table 4). Subsequently, when assessing the discriminative capacity of these four miRNAs to identify pheno-conversion, an area under the curve (AUC) of 0.82 (95% CI: 0.71–0.93) was observed to discern pheno-converted from non-pheno-converted G2019S carriers (Fig. 5). These four miRNAs hold potential as candidate pheno-conversion biomarkers and could be prioritized in subsequent studies.

### Longitudinal RT-qPCR

**Fig. 6:**
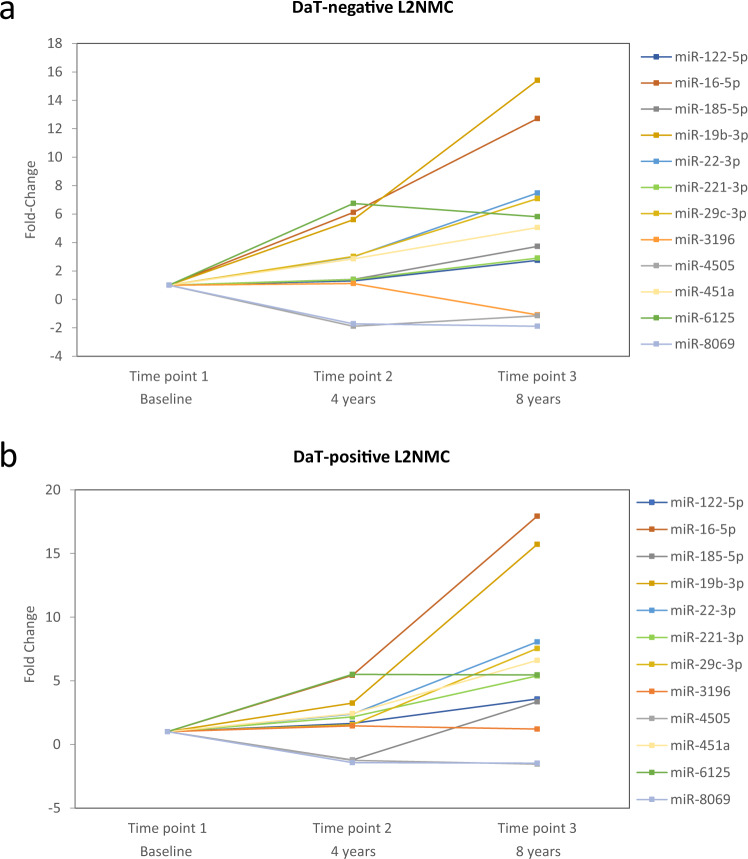
8-year longitudinal follow-up of serum miRNA levels in DaT-negative and DaT-positive L2NMC groups with respect to their corresponding baselines. Longitudinal fold-change values in **a** DaT-negative L2NMC and **b** DaT-positive L2NMC. DaT DaT-SPECT imaging, L2NMC LRRK2 non-manifesting carriers.

To further assess specific miRNA changes across time before motor manifestation, by RT-qPCR, we assessed the longitudinal expression levels of the same 12 cross-sectional miRNA in L2NMC. To this end, we used the DaT-negative and DaT-positive L2NMC cross-sectional serum samples as the reference (baseline)^31^, and compared it to their seriated samples after 4 (time point 2) and 8 years (time point 3) of follow-up. Compared to their respective baselines, longitudinal time points 2 and 3 from DaT-negative and DaT-positive L2NMC^20^ showed significant miRNA variation for 11 of the 12 studied miRNAs (Table 3 and Fig. 6). More specifically, 5 DEmiR showed continued expression variation which was consistent across both follow-up time points in DaT-negative and DaT-positive L2NMC (miR-16-5p, miR-19-5p, miR-22-3p, miR-451a, and miR-6125), indicating longitudinally sustained expression changes for some miRNAs. MiR-8069 was deregulated in the longitudinal assessment only in DaT-negative L2NMC but not in the DaT-positive group, thus suggesting early deregulation prior to DaT-SPECT decline for this miRNA. Other miRNAs showed expression variations in only one of the two follow-up time points (miR-122-5p, miR-185-5p, miR-221-3p, miR-29c-3p, and miR-4505) and require further investigation. Lastly, miR-3196, which was found differentially expressed in all G2019S carriers groups including L2PD in the cross-sectional analysis, did not show L2NMC longitudinal changes compared to baseline, thus indicating that the deregulation of miR-3196 was steady across all G2019S carrier stages and did not depend on DaT-SPECT status. Conversely, miR-122-5p was differentially expressed in the L2PD validation group but also in time point 3 from both DaT-positive and DaT-negative L2NMC of the longitudinal analysis, potentially suggesting dynamic levels of this miRNA which need further investigation. Altogether, we observed that, compared to DaT-negative and DaT-positive L2NMC baselines, the expression levels of miRNAs deregulated at PD premotor stages are dynamic and can vary across time, at least during the 8-year follow-up period of this study.

**Table 3:** Longitudinal follow-up of serum miRNA levels during 8 years in DaT-negative and DaT-positive L2NMC with respect to their baselines.

	DaT-negative L2NMC				DaT-positive L2NMC			
MicroRNA	Time point 2 (*n* = 18)		Time point 3 (*n* = 16)		Time point 2 (*n* = 16)		Time point 3 (*n* = 13)	
	FC	Adj. *p*	FC	Adj. *p*	FC	Adj. *p*	FC	Adj. *p*
miR-122-5p	1.30	0.5467	2.74	0.0117	1.66	0.3585	3.57	0.0190
miR-16-5p	6.12	4.0 × 10^−6^	12.71	<1.0 × 10^−6^	5.43	0.0002	17.94	<1.0 × 10^−6^
miR-185-5p	1.40	0.1996	3.73	0.0002	−1.22	0.5354	3.35	0.0005
miR-19b-3p	5.60	<1.0 × 10^−6^	15.40	<1.0 × 10^−6^	3.25	0.0055	15.72	<1.0 × 10^−6^
miR-22-3p	2.99	3.5 × 10^−5^	7.48	<1.0 × 10^−6^	2.39	0.0087	8.06	<1.0 × 10^−6^
miR-221-3p	1.39	0.1996	2.90	0.0005	2.16	0.0273	5.39	2.0 × 10^−6^
miR-29c-3p	3.02	0.0001	7.09	<1.0 × 10^−6^	1.54	0.2551	7.55	1.0 × 10^−6^
miR-3196	1.11	0.5663	−1.09	0.5548	1.46	0.1274	1.21	0.3083
miR-4505	−1.89	0.0015	−1.16	0.4436	−1.25	0.0068	−1.54	0.1144
miR-451a	2.85	0.0017	5.05	2.5 × 10^−5^	2.42	0.0273	6.60	3.0 × 10^−6^
miR-6125	6.75	<1.0 × 10^−6^	5.81	1.0 × 10^−6^	5.51	0.0002	5.46	7.0 × 10^−6^
miR-8069	−1.72	0.0004	−1.89	0.0006	−1.43	0.0879	−1.47	0.0709

RT-qPCR analysis comparing time point 2 and 3 of DaT-negative and DaT-positive L2NMC groups with their respective baselines.

*DaT* DaT-SPECT imaging, *L2NMC* LRRK2 non-manifesting carriers, *L2PD* LRRK2-associated PD patients, *iPD* idiopathic PD patients, *FC* fold change, *Adj. p* FDR multiple-test adjusted *p* value.

### Biological enrichment analysis

Lastly, we performed a biological enrichment analysis exploring the functionality of only experimentally validated genes from the miRTarbase, which are described to be targeted by the candidate miRNAs associated with the G2019S mutation. To that end, we used the top 10 miRNAs from each comparison from the discovery study. We found a higher number of target genes in iPD patients than in G2019S carriers, and iPD was also the only group surpassing the FDR-multiple testing adjustment (Table 4). Enriched biological functions in iPD differed from G2019S carriers and included gene expression, AKT, TGFb signaling, and senescence/TP53. In G2019S carriers, top deregulated pathways included Rho GTPases in DaT-negative and DaT-positive L2NMC and NOTCH signaling in DaT-positive L2NMC and L2PD. These pathways identified in G2019S carriers are involved in dendritic development and axonal arborization and have been extensively associated with LRRK2 biological functions before^32–34^.

**Table 4:** Biological enrichment analysis of experimentally validated target genes from the top 10 DEmiR from discovery analysis in all main cross-sectional study groups.

Reactome—DaT-negative G2019S L2NMC	*p* value	Adj. *p*
Disinhibition of SNARE formation	0.0009	1
MECP2 regulates neuronal receptors and channels	0.0093	1
Response to elevated platelet cytosolic Ca2+	0.0103	1
Regulation of KIT signaling	0.0105	1
Signaling by Rho GTPases	0.0120	1
Rho GTPase effectors	0.0127	1
Signaling by ERBB2	0.0130	1
Innate immune system	0.0140	1
Antimicrobial peptides	0.0153	1
Axon guidance	0.0196	1
166 user IDs/165 user IDs unambiguously mapped to 165 unique EntrezGene IDs/1 unmapped		
Reactome—DaT-positive G2019S L2NMC	*p* value	Adj. *p*
Signaling by EGFR	0.0104	1
Collagen degradation	0.0221	1
Signaling by NOTCH1	0.0249	1
Constitutive signaling by NOTCH1	0.0249	1
Release of Hh-Np from the secreting cell	0.0285	1
Regulation of cytoskeletal remodeling and cell spreading by IPP complex	0.0285	1
Removal of aminoterminal propeptides from γ-carboxylated proteins	0.0354	1
GAB1 signalosome	0.0389	1
RHO GTPases activate KTN1	0.0389	1
γ-carboxylation, transport, and aminoterminal cleavage of proteins	0.0389	1
65 user IDs/65 user IDs unambiguously mapped to 65 unique EntrezGene IDs		
Reactome—G2019S L2PD	*p* value	Adj. *p*
Estrogen-dependent gene expression	0.0006	0.5460
ESR-mediated signaling	0.0006	0.5460
Signaling by nuclear receptors	0.0013	0.7380
Pre-NOTCH transcription and translation	0.0048	0.8148
RUNX1 genes/megakaryocyte differentiation and platelet function	0.0052	0.8148
RUNX1 regulated expression of components of tight junctions	0.0057	0.8148
RUNX1 regulated transcription of genes involved in interleukin signaling	0.0057	0.8148
Pre-NOTCH expression and processing	0.0065	0.8148
Senescence-associated secretory phenotype	0.0066	0.8148
Generic transcription pathway	0.0067	0.8148
24 user IDs/24 user IDs unambiguously mapped to 24 unique EntrezGene IDs		
Reactome—iPD	*p* value	Adj. *p*
Gene expression (transcription)	7.25E−13	1.25E−09
Generic transcription pathway	2.16E−11	1.87E−08
RNA polymerase II transcription	9.32E−11	5.37E−08
Transcriptional regulation by TP53	3.87E−08	1.67E−05
Oncogene induced senescence	4.89E−08	1.69E−05
Signaling by TGF-beta family members	3.80E−07	1.09E−04
PIP3 activates AKT signaling	5.40E−07	1.33E−04
Circadian clock	1.40E−06	3.02E−04
Intracellular signaling by second messengers	1.77E−06	3.05E−04
Cellular responses to external stimuli	1.95E−06	3.05E−04
1150 user IDs/1139 user IDs unambiguously mapped to 1139 unique EntrezGene IDs/11 unmapped		

*DaT* DaT-SPECT imaging, *L2NMC* LRRK2 non-manifesting carriers, *L2PD* LRRK2 carrier with symptomatic Parkinson disease, *iPD* idiopathic PD, *Adj. p* FDR multiple-test adjusted *p* value.

## Discussion

The lack of informative biomarkers hampers the prediction of disease progression in G2019S L2NMC^35^. Here, we profiled serum miRNAs in a cohort of G2019S carriers from Spain stratified by DaT-SPECT. We observed dynamic miRNA expression profiles across the continuum of progression stages and identified 8 validated DEmiR in DaT-negative L2NMC, 6 in DaT-positive L2NMC, and one in L2PD. The strongest miRNA deregulation was in asymptomatic G2019S carriers, occurred along with the progressive DaT-SPECT decline before motor manifestation, and seemed to attenuate after the diagnosis of L2PD. Moreover, by an 8-year follow-up of DaT-negative and DaT-positive L2NMC, we detected sustained longitudinal variation of 11 DEmiR compared to their baselines. Our study identified specific miRNA changes in PD prodromal stages before motor manifestation, especially in DaT-positive L2NMC who are at a higher risk of PD compared to the general population. Moreover, we identified 4 miRNAs with a reasonable discriminative power to discern pheno-converted vs. non-pheno-converted G2019S carriers, which hold promise as early progression biomarkers for L2PD.

In iPD, we found stronger miRNA deregulation than in G2019S carriers and a higher number of miRNA-targeted genes. These findings are relevant given that L2PD is used to model iPD based on their similar clinical features^36^, yet with a certain degree of motor and non-motor heterogeneity^37^ and a slower progression described for L2PD^38,39^. Thus, at the molecular level, we also found different miRNA profiles between L2PD and iPD with a total of 46 DEmiR. As a possible interpretation, LRRK2 has been shown to directly interact with the Argonaute-2 component of the RNA-induced silencing complex (RISC)^40,41^. Therefore, unlike iPD, miRNA deregulation in LRRK2 carriers could be partially mediated by changes in the miRNA machinery related to the pathogenic activity of mutant LRRK2. Similarly, two earlier studies showed distinct miRNA fingerprints in iPD and monogenic PD patients with *LRRK2*, *SNCA*, or *GBA* mutations^31,42^. In addition, miR-19b-3p and miR-29c-3p were previously associated with iPD^25,26,29,30,43,44^ or idiopathic RBD^27,28^, and were also deregulated in our study only in iPD but not in G2019S carriers. Altogether, these results align with previous findings^31,42^ indicating group-specific miRNA deregulation between iPD and L2PD and an overall greater miRNA deregulation effect in iPD than in L2PD, which is compliant with the complex multifactorial etiology of iPD^45,46^.

Ours is the first study addressing miRNA changes in PD premotor stages, i.e., G2019S carriers with and without DaT-SPECT alterations, during an extended follow-up of 8 years. In G2019S carriers, we found that miRNA changes were more prominent in L2NMC than L2PD, suggesting an attenuation of miRNA deregulation after the diagnosis of PD. Specifically, we found 7 DEmiR deregulated in L2NMC both cross-sectional and longitudinally (miR-122-5p, miR-16-5p, miR-185-5p, miR-451a, miR-4505, miR-221-3p, and miR-8069). Apart from miR-8069, which is novel, six of these miRNAs were earlier described in PD patients with overt disease. Thus, a transcriptome study identified miR-4505 in L2PD blood^47^. Another RNA-seq study using PPMI cohorts found altered miR-122-5p, miR-16-5p, miR-185-5p, and miR-451a in monogenic PD with *LRRK2*, *SNCA*, or *GBA* mutations, and also, miR-16-5p and miR-451a in iPD^31^. Lastly, miR-185-5p and miR-221-3p are part of a 5-miRNA panel discriminating iPD serum from controls^48^. Collectively, these studies support the potential of miRNAs from our study as candidate biomarkers of early PD progression before motor manifestation.

In light of our findings, one hypothesis is that the G2019S pathogenic mutation, which leads to a toxic gain of kinase function of LRRK2^49^, is also associated with miRNA deregulation processes in early premotor stages, which attenuate after motor manifestation in L2PD. Other studies described early molecular deregulation in subjects at-risk of PD before the clinical diagnosis. Thus, several reports showed a higher transcriptomic deregulation in L2NMC than in L2PD^47^ (L2NMC/L2PD ratio of 1.64) or in PD prodromal stages than in manifested PD (L2NMC/L2PD ratio of 2.36)^50^. In our longitudinal miRNA assessment of DaT-negative and DaT-positive L2NMC, we also found progressive miRNA variation after 4 and 8 years compared to their respective baselines. These results align with a 3-year longitudinal RNA-seq study in a PPMI cohort including iPD and monogenic L2PD reporting dynamic miRNA levels across the progression of PD^31^. Similarly, our 8-year longitudinal follow-up in G2019S carriers indicates dynamic miRNA expression changes in premotor stages of PD.

Overall, exploring the prodromal stage of PD is essential to elucidate early disease mechanisms and identify early PD progression biomarkers^51,52^. Illustratively, a recent cerebrospinal fluid (CSF) study reported that levels of total and oligomeric α-synuclein and TNF-α discriminate L2NMC from L2PD and iPD, and controls^53^, thus proposing these candidates as risk biomarkers in prodromal PD stages. Moreover, another study has recently identified specific phospho-protein changes associated with the G2019S mutation in L2NMC^54^. Similarly, here we found specific miRNA deregulation in G2019S carriers with and without DaT-SPECT decline before the diagnosis of PD. Thus, as earlier suggested^54,55^, a relevant implication of our findings is that initiation of LRRK2 inhibitors treatment in L2NMC before motor manifestation represents an attractive option to investigate in clinical trials. In this context, miRNAs detected in L2NMC before motor manifestation warrant further investigation as candidate early progression biomarkers. Specifically, we identified a combination of 4 miRNAs (miR-4505, miR-8069, miR-6125, and miR-451a) that exhibits an AUC of 0.82 in discriminating 8-year followed-up non-pheno-converted DaT-positive L2NMC from pheno-converted L2PD patients. Hence, these 4 miRNAs hold potential as candidate pheno-conversion biomarkers and could be prioritized in subsequent longitudinal studies on *LRRK2* G2019S carriers or other prodromic PD cohorts.

Lastly, we performed a restrictive functional enrichment analysis using experimentally validated target genes only from the top 10 deregulated miRNAs from each discovery group. We found that the biological enrichment in iPD was substantially different from G2019S carriers and targeted the AKT and TGF-beta pathways, which are mostly related to the immune system and inflammation^56,57^. In all G2019S carriers, we found consistent deregulation of Rho GTPases and NOTCH signaling. These pathways play a key role in axonal guidance by regulating neural arborization and dendritic development and are involved in neurodegenerative processes^58,59^. Moreover, the Rho GTPase RAC1 and NOTCH are functionally related to LRRK2, and can be affected by *LRRK2* mutations^32,33^. By mass-spectrometry phospho-proteome analysis, Rho GTPases were also reported among the top deregulated pathways in peripheral blood cells from G2019S carriers^54^. Lastly, Rho GTPases and NOTCH have also been proposed as therapeutic targets for PD^58,60^. Altogether, these studies further strengthen a plausible role for miRNA deregulation in G2019S carriers.

Despite some exciting observations on differential miRNA expression profiles in L2NMC, our study has limitations. First, we performed an extensive longitudinal study of L2NMC characterized by DaT-SPECT, but did not longitudinally followed-up L2PD, iPD, or controls. Second, during our 8-year follow-up period, only 4 out of 20 DaT-positive L2NMC (20%) pheno-converted; therefore, more extended follow-up periods are needed in future studies. Third, given that not all L2NMC are expected to develop PD, our study did not explore other risk or protective factors modulating the penetrance of mutations in *LRRK2* beyond miRNAs. Fourth, no other PD-associated mutations other than in *LRRK2* gene were screened. Lastly, a limitation of many miRNA studies is the inter-lab variability regarding biospecimens, techniques, and normalizers and the lack of consensus guidelines, thus eventually limiting cross-lab reproducibility.

In summary, our study identifies novel miRNA alterations co-occurring with progressive DaT-SPECT decline in premotor stages of PD before the diagnosis of PD. If validated, some of the identified miRNAs hold potential as early progression biomarkers or pheno-conversion in premotor G2019S carriers. These findings may have implications for early PD detection or early neuroprotective strategies when available.

## Methods

### Subjects

All subjects provided written informed consent, and the Ethics Committees of IDIBAPS-Hospital Clínic de Barcelona and Hospital Marqués de Valdecilla approved the study. Serum samples and clinical data from probands were collected at the Movement Disorder units from Hospital Clínic de Barcelona and Hospital Marqués de Valdecilla in Santander (Table 1). By center and group, our cohort included *n* = 20 DaT-negative G2019S L2NMC (7 from Barcelona/13 from Santander), *n* = 20 DaT-positive G2019S L2NMC (7/13), *n* = 20 G2019S L2PD patients (7/13), *n* = 19 iPD cases (7/12), and *n* = 40 healthy controls (27/13). We additionally recruited *n* = 19 L2PD patients and *n* = 20 controls, all from Barcelona, to further assess miRNA findings in a second L2PD cohort (total studied *n* = 158). PD patients were clinically diagnosed according to the UK PD Society Brain Bank criteria, except for the fact that more than one affected relative with PD was not an exclusion criterion^61^. We genotyped all LRRK2 G2019S carriers and iPD patients for the LRRK2 G2019S and R1441G/C/H mutations using commercial Taqman SNP assays-on-demand on a StepOnePlus™ Real-time PCR System. No systematic sequencing was performed to exclude additional mutations causing monogenic PD. We characterized G2019S L2NMC by DaT-SPECT imaging using 123I-2β-carbomethoxy-3β-(4-iodophenyl)-N-(3-fluoropropyl)-nortropane (123I-FP-CIT) SPECT. Images were acquired in a dual‐headed gamma camera (E‐Cam, Siemens). L2NMC were accordingly dichotomized into DaT-negative (normal) and DaT-positive (abnormal dopaminergic function) SPECT status. For longitudinal assessment of L2NMC, subjects were clinically followed-up on average every 2 years and, beyond the baseline, donated two additional serum samples, thus covering a follow-up period above 8 years. In this period, 4 DaT-positive L2NMC (20%) from Santander developed motor symptoms and met the criteria for clinical PD diagnosis.

### Serum miRNA isolation

A total of 5 ml peripheral blood was collected in tubes without anticoagulant (BD Vacutainer) and centrifuged 10 min at 1500 × *g* and 4 °C. Serum was aliquoted into polypropylene CryoTubes (Greiner Bio-One) and kept at −80 °C until use. Total miRNA enriched RNA was extracted using the miRNeasy Serum/Plasma Kit (QIAGEN #217184). As a carrier for miRNA isolation, we added 2 ul of diluted yeast tRNA (Invitrogen #AM7119) into 200 ul of serum for a final concentration of 10 ug/ml. RNA concentration was determined for all samples on a NanoDrop ND-3300 fluorospectrometer (Thermo Fisher Sci.), and miRNA quality was evaluated for a subset of randomly selected samples by electropherogram using an Bioanalizer Small RNA Kit (Agilent #5067-1548).

### Genome-wide miRNA analysis

In the discovery genome-wide miRNA expression analysis, serum miRNA samples were hybridized individually and blind to operator for 42 h onto Affymetrix GeneChip miRNA 4.0 Array (Applied Biosystems #902411; product datasheet), which contains probes for more than 4603 human miRNAs (2578 mature miRNAs and 2025 pre-miRNAs). Images were scanned using an Affymetrix GeneChip Scanner 3000 7G. Files generated by the Affymetrix GeneChip Command Console (AGCC) were processed with the Expression Console software to determine the data quality. Microarray raw data were analyzed using Partek Genomic Suite v7.0 software applying the robust multi-array average (RMA) background correction model, which allows the relative comparison of miRNA abundance in different arrays. Only miRNAs with fluorescence detection values above 2.4 arbitrary units and a significant expression *p* value below 0.05 compared to background were considered expressed in human serum samples. In line with previous reports, up to 10% of the mature human miRNA screened in the array were expressed in human serum^62^. For differential miRNA expression analysis, we used the global normalization method adjusting by sex, age, and hybridization date. We compared the miRNA expression levels of DaT-negative L2NMC, DaT-positive L2NMC, L2PD, or iPD with controls, or among study groups (DaT-negative vs. DaT-positive L2NMC, DaT-positive L2NMC vs. L2PD, and L2PD vs. iPD) in a two-tailed Student’s *t* test. We defined as candidate differentially expressed miRNAs for subsequent RT-qPCR assessment those miRNAs with a significance double criterion of a fold-change above |1.5| and a *p* value below 0.05.

### RT-qPCR miRNA analysis

Serum miRNA samples were reverse-transcribed and pre-amplified using TaqMan Advanced miRNA cDNA Synthesis Kit (Thermo Fisher Sci. #A28007) in a Veriti^TM^ 96-well Thermal Cycle (Applied Biosystems #4375786). For each miRNA, cDNA pre-amplified products were quantified using TaqMan Fast Advanced Master Mix (Thermo Fisher Sci. #4444557) and TaqMan Advanced miRNA Assays (Thermo Fisher Sci. #A25576). Reactions were performed per duplicate on a TaqMan® StepOnePlus™ Real-Time PCR System (Applied Biosystems) using 96-well real-time PCR plates at a final volume of 10 µl. After discarding commercially available assays which did not amplify at a minimum quality level in serum samples, by real-time quantitative PCR (RT-qPCR) we tested a total of 12 candidate miRNAs. These included 10 candidate DEmiR detected by the array (miR-122-5p, miR-16-5p, miR-185-5p, miR-19b-3p, miR-22-3p, miR-221-3p, miR-29c-3p, miR-3196, miR-4505, miR-451a, miR-6125 and miR-8069), and 2 additional candidate miRNA earlier reported in iPD (miR-29c-3p)^25,27^ or well-established PD prodromal stages (idiopathic RBD) (miR-451a)^28^. Among the candidate miRNAs selected from the array, miR-185-5p, miR-6125, miR-3196, miR-4505, and miR-22-3p were detected in more than one comparison (Supplementary Table 1), and miR-19b-3p and miR-221-3p were previously reported as deregulated miRNAs in PD^25^ and idiopathic RBD^28^. For normalization, we selected miR-320a-3p and miR-6727-5p as endogenous references. These miRNAs were unbiasedly nominated among the miRNAs showing the most stable expression across all samples using the NormFinder software^63^. For relative quantification, we applied the ∆∆*C*_T_ method using the DataAssist v3.0 software (Applied Biosystems). The maximum allowable *C*_T_ value was set at 35. Statistical significance levels for RT-qPCR validated differentially expressed miRNAs (DEmiR) were established at a fold-change difference above |1.5|, and a Benjamini-Hochberg FDR adjusted *p* value below 0.05 under a two-tailed Student’s *t* test.

### ROC analysis

To infer the probability of each patient to undergo pheno-conversion based on miRNA expression values, logistic regression models were fitted by using the *caret* framework in R, and including leave-one-out cross-validation to reduce overfitting of predicted data. The discriminative ability of the differentially expressed miRNAs was assessed by means of the area under the curve (AUC) on a receiver operating characteristic (ROC) curve employing the *pROC* package.

### Biological enrichment analysis

As a functional analysis, we selected only the 10 top miRNAs identified in each genome-wide discovery group as compared to controls and performed a miRNA target gene analysis using the Mienturnet tool (link). Using the miRTarbase database, we further filtered-in only experimentally validated target genes which were targeted by one or more miRNAs. Lastly, we performed a biological enrichment analysis using the pathway database Reactome in Webgestalt online source (link) under the significance cut-off of a Benjamini-Hochberg FDR adjusted *p* value below 0.05.

### Reporting summary

Further information on research design is available in the Nature Research Reporting Summary linked to this article.

## Supplementary information

Reporting Summary

Supplementary tables

## Data Availability

The GeneChip miRNA array data are available at the genome expression omnibus (GEO) under the accession code GSE221543.
